# Calcium Uptake and Proton Transport by Acidocalcisomes of *Toxoplasma gondii*

**DOI:** 10.1371/journal.pone.0018390

**Published:** 2011-04-25

**Authors:** Peter Rohloff, Kildare Miranda, Juliany C. F. Rodrigues, Jianmin Fang, Melina Galizzi, Helmut Plattner, Joachim Hentschel, Silvia N. J. Moreno

**Affiliations:** 1 Department of Cellular Biology and Center for Tropical and Emerging Global Diseases, University of Georgia, Athens, Georgia, United States of America; 2 Instituto de Biofisica Carlos Chagas Filho, Universidade Federal do Rio de Janeiro, Rio de Janeiro, Rio de Janeiro, Brazil; 3 Fachbereich Biologie, Universitaet Konstanz, Konstanz, Germany; Institut national de la santé et de la recherche médicale - Institut Cochin, France

## Abstract

Acidocalcisomes are acidic calcium stores found in diverse organisms, being conserved from bacteria to humans. They possess an acidic matrix that contains several cations bound to phosphates, which are mainly present in the form of short and long polyphosphate chains. Their matrix is acidified through the action of proton pumps such as a vacuolar proton ATPase and a vacuolar proton pyrophosphatase. Calcium uptake occurs through a Ca^2+^/H^+^ countertransporting ATPase located in the membrane of the organelle. Acidocalcisomes have been identified in a variety of microorganisms, including Apicomplexan parasites such as *Plasmodium* and *Eimeria* species, and in *Toxoplasma gondii*. We report the purification and characterization of an acidocalcisome fraction from *T. gondii* tachyzoites after subcellular fractionation and further discontinuous iodixanol gradient purification. Proton and calcium transport activities in the fraction were characterized by fluorescence microscopy and spectrophotometric methods using acridine orange and arsenazo III, respectively. This work will facilitate the understanding of the function of acidocalcisomes in Apicomplexan parasites, as we can now isolate highly purified fractions that could be used for proteomic analysis to find proteins that may clarify the biogenesis of these organelles.

## Introduction

Acidocalcisomes are acidic organelles rich in calcium and other cations bound to polymers of phosphate. They are present in a wide variety of cells from bacteria to human and are best characterized in trypanosomatids [1]. Acidocalcisome-like organelles appear to be present in *Toxoplasma gondii*, the etiological agent of toxoplasmosis, and several research reports have shown a preliminary characterization: (1) electron microscopy studies have demonstrated the presence of the typical electron-dense organelles which have X-ray spectra consistent with the presence of high concentrations of phosphorus together with calcium, magnesium, sodium, potassium, and zinc [2]; (2) biochemical and immunological evidence is consistent with the presence of a Ca^2+^-ATPase (TgA1) [3] and a vacuolar-type H^+^-pyrophosphatase (TgVP1) [4] in these organelles, and polyphosphatase and bafilomycin A_1_-sensitive ATPase activities could be detected in an organelle-enriched fraction; (3) DAPI staining of intact cells and biochemical analysis of organelle-enriched fractions revealed the presence of pyrophosphate (PP_i_) and short- and long-chain polyphosphate (poly P) [2]. Furthermore, addition of ionophores (ionomycin), V-H^+^-ATPase inhibitors like bafilomycin A_1_, or NH_4_Cl to intact tachyzoites triggered hydrolysis of poly P in parallel to an increase in intracellular Ca^2+^ concentration [2], [5], suggesting a close association between poly P hydrolysis and Ca^2+^ release from an acidic compartment of characteristics similar to those of acidocalcisomes of other microorganisms [1], [6]; (4) Finally, a link between calcium homeostasis, invasion, virulence, and poly P content was pointed out by the phenotypic analysis of *T. gondii* mutants deficient in TgA1, which showed decreased PP_i_ and poly P levels together with alterations in intracellular Ca^2+^ homeostasis, microneme secretion, invasion, and virulence [7].

However, despite these preliminary studies, several biochemical properties of the acidocalcisomes of *T. gondii* remain to be defined. For example, based on the pH-dependent accumulation of 3-(2,4-dinitroanilino)-3′amino-N-methyldipropylamine (DAMP), as detected by electron microscopy, it has been suggested that the only acidic compartments in *T. gondii* tachyzoites are the mature and forming rhoptries [8]. Those results were consistent with earlier light microscopy observations demonstrating the presence of acidic compartments, as detected by acridine orange staining, only in the anterior regions of tachyzoites [9]. Other authors [10] have suggested that as some punctuate background staining has been noted in *T. gondii* using generic antisera against peptides corresponding to *Arabidopsis thaliana* VP1 [11], data on the localization of TgVP1 should be confirmed by other methods. In addition, while Ca^2+^ and H^+^-transport could be demonstrated in isolated acidocalcisomes from trypanosomatids [12], [13] or other microorganisms [14], this was not possible using isolated acidocalcisomes from *T. gondii* [2]. Finally, previous methods for subcellular fractionation and isolation of acidocalcisomes from *T. gondii* have been limited by low yields and contamination with other organelles, most notably mitochondria (Rohloff, P., and S. Moreno, unpublished results), which has made them unsuitable for certain kinds of studies, including detailed biochemical analysis.

Here we report a modified protocol for the purification of *T. gondii* acidocalcisomes, which provides a fraction suitable for biochemical studies and place the quality of evidence for the presence of acidocalcisomes in *T gondii* at the level of that from the initial, definitive characterization in trypanosomatids.

## Materials and Methods

### Culture methods

*T. gondii* tachyzoites of the RH strain and the TgVP1-OE clone were cultivated in human skin fibroblasts and purified as described before [15].

### Chemicals and reagents

Iodixanol (Optiprep) was from Sigma-Aldrich. The enhanced chemiluminescence detection kit was from Amersham Life Sciences, Inc. (Arlington Heights, IL). Aminomethylenediphosphonate (AMDP) was synthesized by Michael Martin in the laboratory of Dr. Eric Oldfield at the Department of Chemistry, University of Illinois at Urbana-Champaign. All other reagents were analytical grade.

### Overexpression of the TgVP1

We isolated tachyzoites that overexpress the vacuolar proton pyrophosphatase by transfecting cells of the RH strain with the plasmid ptubTgVP1-FLAG/sag-CAT, which contains the entire coding sequence of the TgVP1 gene. Selection was done in the presence of chloramphenicol and one clone was chosen for further analysis [17]. These cells (TgVP1-OE) were used, together with the wild-type cells, to measure V-H^+^-PPase activity and for purification of acidocalcisomes. TgVP1-OE had a higher content of TgVP1 as detected by western blot and immunofluorescence analyses [17]. These cells also had approximately 8–10-fold higher pyrophosphate stimulated proton transport activity (Fig. 3, compare the slopes between A and B).

### Isolation of Acidocalcisomes

**Figure 1:**
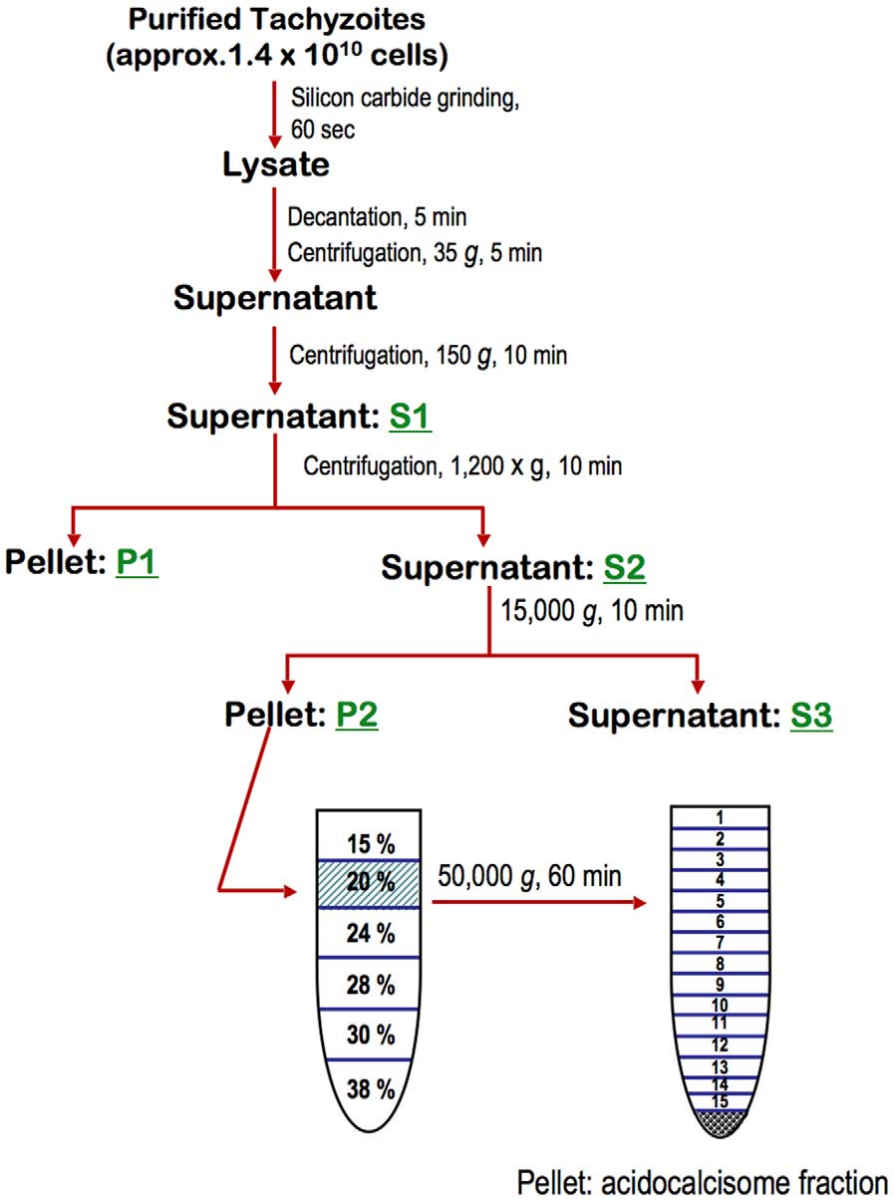
Schematic depiction of the subcellular fractionation method used for the isolation of acidocalcisomes from *T. gondii* tachyzoites. Tachyzoites are homogenized by grinding with silicon carbide for 60 sec and the mixture of broken cells and silicon are re-suspended in lysis buffer. This homogenate is allowed to decant and centrifuged using two low speed steps (35 *g* and 150 *g*) to eliminate contaminating silicon carbide. The supernatant (S1), obtained after the 150 *g* centrifugation is centrifuged at 1,200 *g*, which removes a great percentage of contaminating mitochondria. S2, the supernatant generated after a 1,200 *g* centrifugation, is centrifuged at 15,000 *g.* Pellet 2 (P2) is generated which is resuspended in lysis buffer and mixed with iodixanol and loaded in the gradient as indicated in the figure. Pellet P1 and Supernatant S3 are discarded. The final pellet obtained after a 1 hour centrifugation at 50,000 *g* contains the acidocalcisomes. The protocol is described in more detail under “Materials and Methods”.

Isolation of acidocalcisomes was according to a modification of the previously described method [2] and is schematically depicted in Fig. 1. Tachyzoites (RH or TgVP1-OE) (∼1–2×10^10^ cells) were centrifuged at 500 *g* for 10 min and the cell pellet washed with lysis buffer (125 mM sucrose, 50 mM KCl, 4 mM MgCl_2_, 0.5 mM EDTA, 20 mM K-Hepes, 5 mM dithiothreitol, protease inhibitors cocktail (1∶500), 12 µg/ml DNAse, 12 µg/ml RNAse, and 8 µg/ml nocodazole, pH 7.2). The cell pellet was mixed with 1.8 X wet weight silicon carbide and lysed by grinding with a pestle and mortar for approximately 60 s (lysis was checked by microscopy every 15 s). The mixture of silicon carbide and lysed cells was resuspended in approximately 40 ml of lysis buffer, and decanted, and centrifugation steps were carried out as depicted in Fig. 1. Pellet P2 was resuspended in lysis buffer and gently homogenized with the aid of a Poulter tissue homogenizer. This sample was loaded into the 20% layer of a discontinuous iodixanol gradient (4 ml steps of 15, 20, 25, 30, 34, and 38% iodixanol). The gradient was centrifuged at 50,000 *g* using a Beckman SW 28 rotor for 60 min. 15 fractions of 1.8 ml each were collected in sequence from the top of the gradient. The acidocalcisomal fraction pelleted on the bottom of the tube and was resuspended in lysis buffer (Fig. 1).

### Enzyme Assays

Pyrophosphatase activities were assayed by measuring phosphate release using the malachite green assay [16]. Aminomethylenediphosphonate (AMDP) was used to distinguish between the vacuolar and the soluble activity [2]. Acid phosphatase was assayed by measuring phosphate release from *p*-nitrophenylphosphate [2] in acetate buffer pH 5.0.

PP_i_- and ATP-driven proton transport in acidocalcisome-enriched fractions was measured by changes in the fluorescence of acridine orange at excitation and emission wavelengths of 470 and 526 nm, respectively, using a Molecular Devices Microplate Reader. Fractions were incubated in a 200 µl final volume of a solution which is described in the figure legends (see below), plus 3 µM acridine orange for 3 min prior to the addition of 100 µM PP_i_ or 0.5 mM ATP. Nigericin was used to collapse the membrane potential generated. Each experiment was repeated at least three times with different fractionations, and the figures show representative experiments.

### Fluorescence microscopy

For imaging of isolated acidocalcisome fractions stained with acridine orange, suspensions were incubated in the presence of 130 mM KCl, 20 mM K-HEPES, 2 mM MgCl_2_, pH 7.2 and 100 µM Na-PP_i_. Images were obtained in an Olympus BX60B epifluorescence microscope equipped with a 488 nm excitation filter set. Emission signal (above 500 nm) was detected with a Hamamatzu digital CCD camera (Model C5810) and an image analysis system attached to the microscope. For visualization of poly P, samples were incubated at room temperature with 10 µg DAPI/ml. After 10 min, the samples were wet-mounted on slides and observed by epifluorescence microscopy using an Olympus WIG filter (excitation 380–385 nm; emission >580 nm).

### Electron microscopy

Energy-dispersive X-ray spectra were recorded from the acidocalcisomes present in whole cells adhered to formvar/carbon coated grids. Specimen grids were examined in a LEO 912 Omega scanning transmission electron microscope operating at 80 kV using a tungsten filament in the scanning transmission mode, and spot size was 63 nm. X ray point measurements were collected for 150 sec using a Li-drifted Si-detector (front area 30 mm^2^) equipped with an ATW atmospheric window. Analyses were performed using an Oxford Link ISIS system attached to the microscope. X-ray mappings were acquired in the same microscope as above, in the scanning transmission (STEM) imaging mode, spot size was 40 nm. X rays were collected by the Li-drifted Si-detector. Analyses were performed using a Link multichannel energy analyzer and Link ISIS 3.00 software (Oxford Instruments, Wiesbaden, Germany).

### Western blot analysis

Western blot analysis was done as previously described [17] using 20 µg protein/lane and a dilution of TgCPL of 1∶4,000.

### Calcium uptake

Experiments were performed using the calcium-sensitive dye Arsenazo III as described previously [18], [19] using an Olis-modified Aminco DW2000 dual wavelength spectrophotometer. Reactions were carried out in a 3 ml cuvette at room temperature. Reaction buffer was: 130 mM KCl, 20 mM K-HEPES, 2 mM MgCl_2_, pH 7.2. For experiments involving permeabilized cells, the reaction mixture consisted of 2 ml reaction buffer containing 1×10^8^ cells, 2 µg/ml oligomycin, 2 µg/ml antimycin A, 50 µM EGTA, 40 µM Arsenazo III, 250 µM CaCl_2_, and 16 µM digitonin. For experiments involving isolated acidocalcisomes, reaction mixture was similar except that an aliquot of acidocalcisome suspension (10 µg/ml protein) was added to the cuvette in place of whole cells and digitonin was omitted.

## Results

### Isolation of acidocalcisomes

In unfixed whole cell preparations of tachyzoites subjected to electron microscopy, acidocalcisomes appear as round, electron-dense structures that are distributed both apically and throughout the cell (Fig. S1A and [2]) and that are morphologically indistinguishable from similar structures in trypanosomatids [1]. Elemental mapping of unfixed whole cell preparations shows that these structures are highly enriched primarily in phosphorus, oxygen, and calcium (consistent with high concentrations of poly P and calcium) [20] (Fig. S1B–D), but also other mono- and divalent cations such as potassium, sodium, magnesium and zinc (Fig. S1E–H). The low sulfur detection in the acidocalcisomes suggests low content of cysteine or methionine-containing proteins (Fig. S1I).

**Figure 2:**
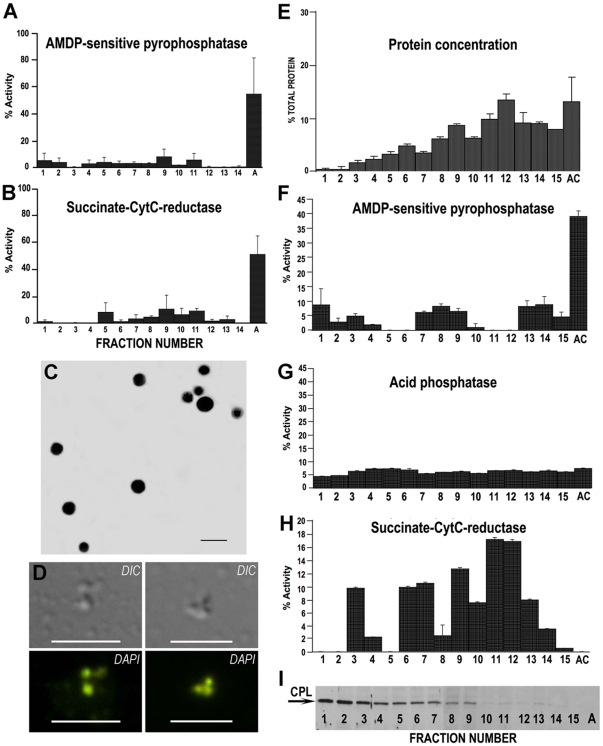
Distribution of markers for various subcellular compartments on iodixanol gradients. Biochemical assays for markers of mitochondria (succinate-cytochrome C reductase), dense granules and rhoptries (acid phosphatase), and acidocalcisomes (AMDP-sensitive pyrophosphatase) were carried out as described in “Materials and Methods.” Enzymatic analyses were made on fractions obtained with the previously published protocol [2] (**A** and **B**) or the presently described (**F–H)** method. Fractions from iodixanol gradients were collected in sequence from the top (Fraction 1) to the bottom (A, acidocalcisomes) of each gradient. This is a representative gradient out of more than 10 fractionations. **D**, acidocalcisomes stain with DAPI and preserve their electron-dense morphology (**C**). A modified DAPI staining protocol was used as described in “Materials and Methods” to detect poly P in isolated acidocalcisomes (DIC and DAPI) by fluorescence microscopy. Isolated acidocalcisomes were also visualized directly by electron microscopy without fixation or sectioning (**C)**. **E** shows the protein concentrations of the fractions obtained after the gradient shown in Fig. 1. **I**, shows the western blot analyses of *T. gondii* Cathepsin L (TgCPL), an plant-like vacuole marker in the different fractions.

We sought to improve on our initial technique to isolate acidocalcisomes from *T. gondii* [2]. This was prompted by our observation that this method was unamenable to scale-up, as attempts to perform large-scale purification of acidocalcisomes routinely resulted in unacceptable levels of contamination with other organelles, primarily mitochondria. As a result, it was unclear whether previous failures to demonstrate proton or calcium translocation in isolated organelles were merely due to the inability to produce sufficient quantities of relatively pure material for biochemical analysis. Our modified method incorporates a longer decantation time after cell lysis, an additional centrifugation step to eliminate most mitochondrial contamination, and changes to the discontinuous iodixanol gradient used in the final ultracentrifugation step. These changes are detailed in Fig. 1 and in “Materials and methods”. When using scaled-up preparations of isolated acidocalcisomes by our previously published protocol [2], the acidocalcisome fraction showed a high level of contaminating mitochondria (Fig. 2B), with greater than 50% of the mitochondrial marker activity, succinate-cytochrome C reductase, co-purifying along with AMDP-sensitive pyrophosphatase (Fig 2A) [21], the acidocalcisome marker [1]. In contrast, following modification, AMDP-sensitive pyrophosphatase remained well concentrated at the bottom of the gradient (Fig. 2F), but without mitochondrial co-enrichment (Fig. 2H). A comparison of the pyrophosphatase activity measured as PP_i_ hydrolysis is presented in Table 1. There is a clear enrichment of the activity in the acidocalcisome fraction when compared to the preceding fractions (Table 1). The enrichment (5.9 and 10-fold when the wild type (WT) or the VP1 overexpressing (TgVP1-OE) (see below) strains was used, Table 1) is not so dramatic because the vacuolar pyrophosphatase also localizes to other acidic compartments such as the recently described plant-like vacuole or PLV [17]. Acidocalcisomes have higher density than the PLV, sediment to the bottom of the iodixanol gradient obtained by fractionating the pellet P2 (Fig. 1) while the PLV fraction is purified from the S3 fraction [17]. Fig. 2I shows that the acidocalcisome fraction (A) is completely devoid of cathepsin L (CPL), a marker for the PLV [17]. Also, relatively low enrichment could be explained by the loss of membrane integrity of a significant portion of purified organelles, as enrichment calculation is based on enzymatic assays. Acid phosphatase, a marker found in rhoptries and dense granules [22], was also not enriched in the acidocalcisome fraction (Fig. 2G). Examination of the resulting acidocalcisome fraction by light microscopy revealed a homogenous population of vesicles which labeled intensely with a poly P-sensitive modified DAPI staining protocol (Fig. 2D, DAPI) and which, when examined by electron microscopy, revealed typical electron-dense acidocalcisome morphology (Fig. 2C).

**Table 1:** Purification of *T. gondii* acidocalcisomes from iodixanol step gradients.

Activity	Strain used	% Yield	Purification-fold
**Pyrophosphatase (AMDP-sensitive)**	WT (RH)	14.20	5.90
	TgVP1-OE	18.50	10.14
**Cytochrome c reductase**	WT (RH)	N.D.*	N.D.*
	TgVP1-OE	0.5	0.3
**Acid phosphatase**	WT (RH)	0.08	0.18
	TgVP1-OE	0.49	0.71
**Pyrophosphatase (AMDP-insensitive)** @	WT (RH)	1.33	0.55
	TgVP1-OE	4.02	2.21

Yield values are percentages relative to the S1 fraction shown in Fig. 1.

*The cytochrome c reductase activity in these fractions were below detectable levels.

# Methods for determination of enzymatic activities are described under “Materials and methods” and in Rodrigues et al, 2002 [2].

Purification fold was calculated from the ratio of the activity in the acidocalcisome fraction to the activity in the S1 fraction (Fig. 1).

@ This activity corresponds to the pyrophosphatase activity insensitive to AMDP. It is used as a cytosolic marker.

AMDP, aminomethylenediphosphonate; WT, wild type cells; TgVP1-OE, *T. gondii* overexpressing vacuolar proton pyrophosphatase.

### Proton pumping activity in isolated acidocalcisomes

Although previous studies have provided biochemical evidence for the presence of important acidocalcisomal markers, such as the vacuolar (AMDP-sensitive) H^+^-pyrophosphatase and the vacuolar (bafilomycin A_1_-sensitive) H^+^-ATPase, in isolated *T gondii* acidocalcisomes [2], it has not been previously possible to demonstrate actual proton translocation in intact organelles. It was postulated that this might have been due either to an effect of the fractionation method or to the inability to retrieve adequate amounts of material for analysis.

**Figure 3:**
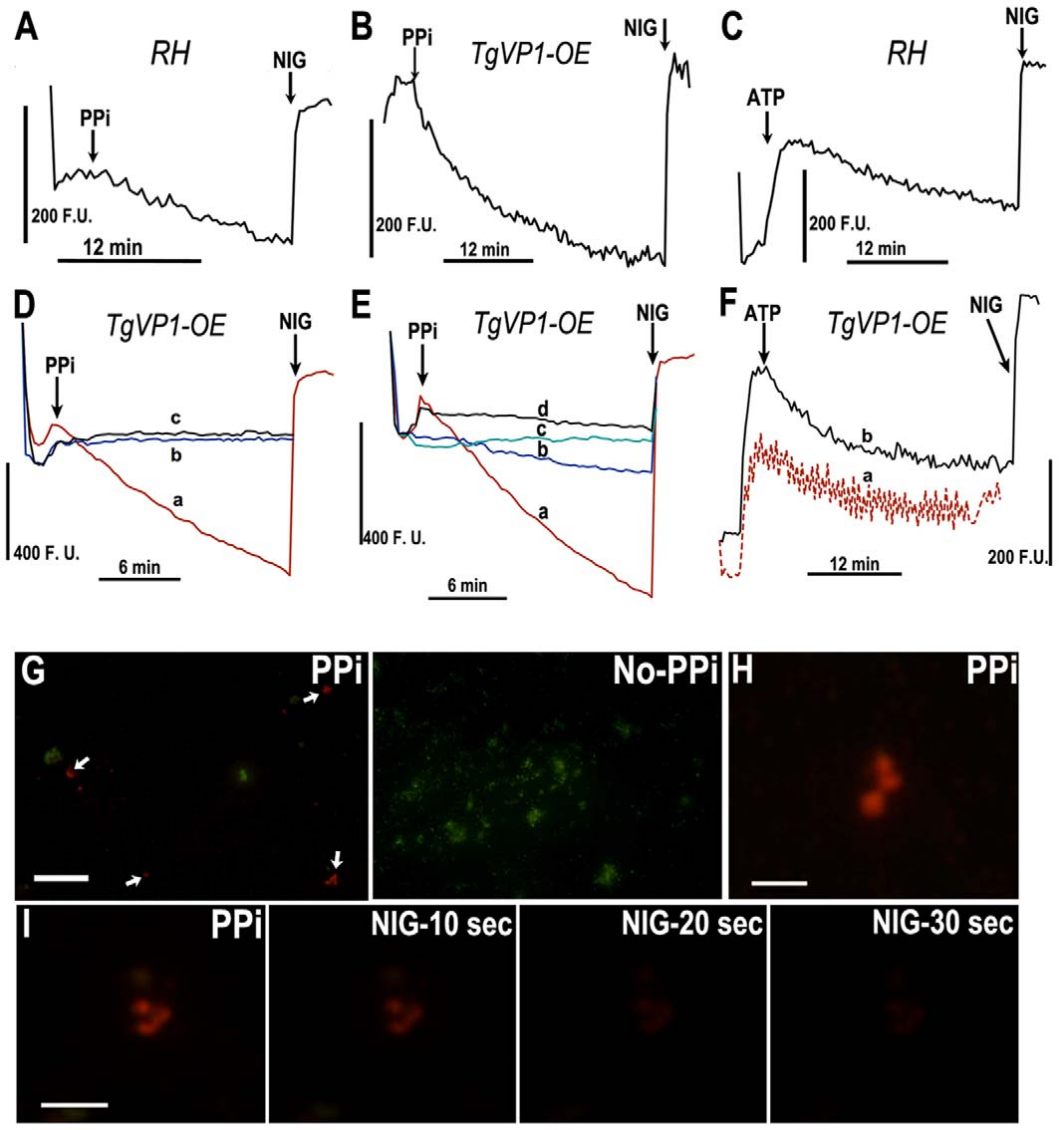
PP_i_ and ATP-driven proton transport in isolated acidocalcisomes of *T. gondii* RH strain and TgVP1-OE cells. The amounts of protein used were: 0.069 mg/ml for **A–C**, and 0.035 mg/ml for **D–F**. The reaction medium contained 130 mM KCl, 2 mM potassium phosphate, 2 mM MgCl_2_, 10 mM Hepes buffer pH 7.2, 50 µM EGTA and 2 µg/ml of both oligomycin and antimycin A. Acridine orange (AO) at 3 µM was added at the beginning of each tracing, and initial fluctuations in the tracing represent dye equilibration. Subsequently, 100 µM PP_i_, 0.5 mM ATP, or 5 µM nigericin (NIG) were added where indicated by the arrows. **A** and **C** are experiments with acidocalcisome fractions obtained after fractionation of RH cells and **B, D, E** and **F** are the results with fractions obtained after fractionation of VP1-OE cells. **D,** AMDP inhibits PP_i_-mediated transport. Traces show proton uptake with PP_i_ alone (*trace a*), after preincubation with 40 µM AMDP followed by PP_i_ (*trace b* **)** and in the absence of PPi (*trace c*). **E,** PP_i_-driven proton transport was measured using different reaction media: 130 mM KCl buffer (*trace a*), 65 mM KCl plus 125 mM sucrose (*trace b*), 250 mM sucrose (*trace c*) and 130 mM NaCl (*trace d*). All buffers contain 2 mM potassium phosphate, 2 mM MgCl_2_, 10 mM Hepes buffer pH 7.2, 50 µM EGTA and 2 µg/mL oligomycin. **F,** Bafilomycin A_1_ (*trace a*) inhibits ATP-mediated proton transport. The reaction medium was as in **A**. The incubation was in the presence (*trace a*) or absence (*trace b*) of 1 µM bafilomycin A_1_. Results shown in **A–F** were qualitatively similar in at least 3 different experiments. **G–I**, Direct visualization of acidocalcisomal acidification by epifluoresence microscopy. Acidocalcisomes from TgVP-OE cells were incubated with acridine orange as detailed under “Materials and Methods” in the presence **(G,** PPi**)** or absence **(G,** no-PPi**)** of PP_i_. Acidified organelles give off a bright orange fluorescence, whereas non-acidic organelles give off a green fluorescence. **H,** shows a typical magnified view of **G**. **I**, After incubation with PP_i_, acidified organelles were incubated with 1 µM nigericin, and fluorescence intensity was followed at 10, 20, and 30 sec. Bars in **G** = 2 µm; **H** = 0.5 µm; **I** = 1 µm. F.U., arbitrary fluorescence units.

We isolated acidocalcisomes from wild-type RH tachyzoites or from a tachyzoite line overexpressing the vacuolar H^+^-pyrophosphatase (TgVP1-OE) [17]. This clone contains higher activity of TgVP1 (1.8 µmols/min/mg protein as measured in a total lysate) than the RH wild type strain (0.3 µmols/min/mg protein) and the enzyme is more stable because it is possible to isolate fractions with high proton transport activity (Fig. 3, compare A with B). These cells grow normally although they always produce lower number of parasites when cultivated in large-scale. PP_i_- or ATP-dependent proton uptake was monitored using acridine orange fluorescence, as described under “Materials and methods.” As shown in Fig. 3, addition of PP_i_ (Figs. 3A, 3B, 3D, 3E) or ATP (Figs. 3C, 3F) to suspensions of isolated acidocalcisomes induced a rapid decrease in the fluorescence of acridine orange at 470–526 nm, corresponding to uptake into acidifying compartments [23]. This was rapidly reversed by collapsing the transmembrane proton gradient using the K^+^/H^+^ exchanger nigericin, confirming that the change in fluorescence was due to organellar acidification. The rate of PP_i_-mediated proton uptake was significantly increased in TgVP1-overexpressing cell lines (Fig. 3B) over the rate in wild type RH cells (Fig. 3A), while the rate of ATP-stimulated uptake was comparable between the fractions obtained from RH (Fig. 3C) or VP1-OE (Fig. 3F) cells. Although the PP_i_-stimulated activity was readily detectable in acidocalcisomes obtained from wild-type cells (Fig. 3A), in the TgVP1-OE acidocalcisomes, the activity was higher and stable for several more hours after isolation; therefore acidocalcisomes from VP1-OE cells were used for further characterization.

To confirm that the ATP- and PP_i_-mediated activities were indeed due to the acidocalcisomal protein components and not contaminants, proton uptake was measured in the presence of AMDP or bafilomycin A_1_, known inhibitors of the acidocalcisomal H^+^-pyrophosphatase and H^+^-ATPase, respectively [1]. PP_i_-mediated uptake was dramatically inhibited by AMDP (Fig. 3D, *trace c*). Similarly, ATP-mediated uptake was reduced by a standard concentration of bafilomycin A_1_ (1 µM), albeit to a lesser degree (Fig. 3F, *trace a*). In addition, no proton uptake was observed without PP_i_ or ATP in the reaction buffer (Fig. 3D, *trace b*). Additionally, as another known characteristic of the H^+^-pyrophosphatase is its significant potassium dependence [24], PP_i_-mediated uptake was measured in the presence of various concentrations of K^+^-containing buffers. Fig. 3E shows the proton transport activity with different buffers containing various concentrations of potassium. Maximum transport activity was obtained with 130 mM potassium (Fig. 3E, trace a) and the activity was lower with lower concentrations of potassium (Fig. 3E, traces b–d).

Acidification of acidocalcisomes, as indicated by changes in the acridine orange fluorescence signal, could also be visualized directly by epifluorescence microscopy. Aliquots of isolated acidocalcisomes were uniformly green without the addition of PP_i_ (Fig. 3G, no-PPi), however addition of PP_i_ induced a change toward orange fluorescence (Fig. 3G, PPi). This change was not uniform, which probably reflects previous observations that a certain percentage of organelles lose much of their functionality, during the isolation procedure [12]. Additionally, collapse of the proton gradient, and loss of orange fluorescence, could be followed in time after the addition of nigericin (Fig. 3I, PPi, NIG-10 sec, NIG-20 sec and NIG-30 sec).

**Figure 4:**
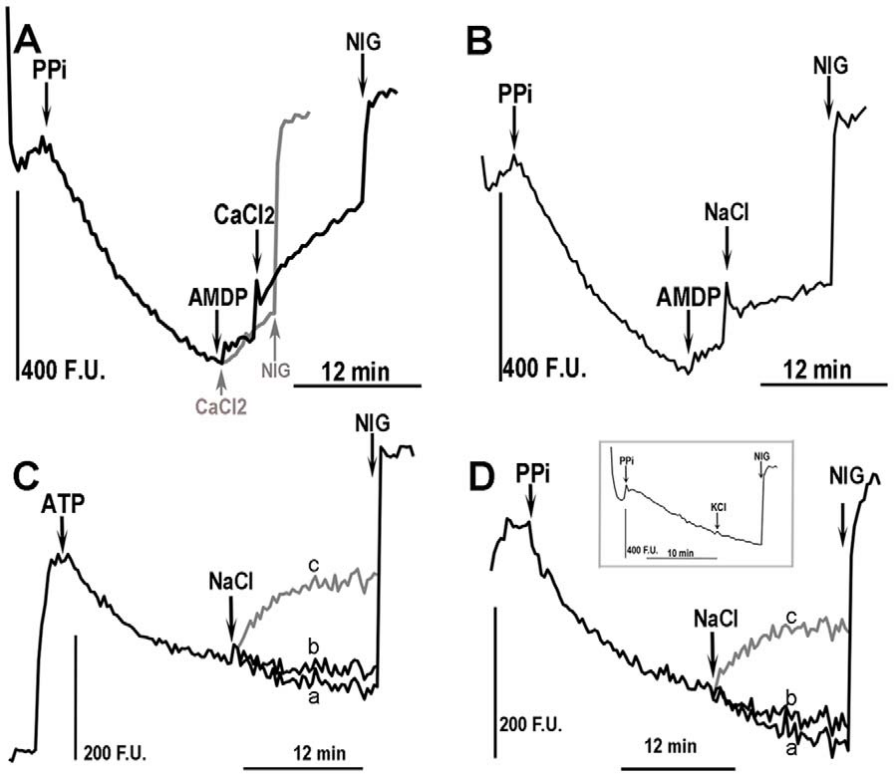
Effects of CaCl_2_ and NaCl on proton efflux in isolated acidocalcisomes of *T. gondii* tachyzoites overexpressing VP1. Reaction medium contained 130 mM KCl, 2 mM potassium phosphate, 2 mM MgCl_2_, 10 mM Hepes buffer pH 7.2, 50 µM EGTA and 2 µg/ml of both oligomycin and antimycin A. Acridine orange (AO) at 3 µM was added at the beginning of each tracing. **A,** proton transport stimulated by 100 µM PP_i_ and inhibited by the addition of 40 µM AMDP (*black tracing*). The addition of 100 µM CaCl_2_ produces release of protons either when added after AMDP (*black tracing)* or directly after PP_i_ (*grey tracing*). **B,** PP_i_-stimulated proton transport is inhibited by AMDP and further addition of 100 mM NaCl releases protons. **C,** proton transport stimulated by 1 mM ATP and released by the addition of 100 mM NaCl (*trace c*). *Trace a* shows a control experiment without any further addition and *trace b* after adding 40 mM NaCl. **D,** proton transport stimulated by 100 µM PPi and released by the addition of 100 mM NaCl (*trace c*). *Trace a* shows a control experiment without any further addition and *trace b* after adding 40 mM NaCl. The inset shows a control experiment using KCl. Nigericin (NIG, 5 µM) was added where indicated by the arrows. F.U., arbitrary fluorescence units. Results shown in **A–D** were qualitatively similar in at least 3 different experiments.

Acidocalcisomes have been studied most extensively in trypanosomatids, and in these organisms there is physiological evidence for the existence of Ca^2+^/H^+^ and Na^+^/H^+^ antiport systems [13], [25]. Therefore, we investigated the effects of sodium and calcium on the acidification of isolated acidocalcisomes. Following PP_i_-induced uptake of acridine orange, and subsequent inhibition of the uptake mechanism with AMDP, addition of either 100 µM CaCl_2_ or 100 mM NaCl induced a rapid reversal of acidification (Figs. 4A and 4B, respectively), consistent with the presence of both of these exchange mechanisms. AMDP was used to inhibit the V-H^+^-PPase and better visualize proton release. However, this effect was also observed after adding CaCl_2_ or NaCl directly after PP_i_ (Figs. 4A, grey trace, and 4D, trace c, respectively). The effect of NaCl (Fig. 4C and 4D) was dose-dependent. The effect of sodium was observed when the acidification was stimulated by either ATP (Fig. 4C) or PP_i_ (Fig. 4D) indicating that it is independent of the source of energy for acidification. A control experiment with KCl is shown in the inset of Fig. 4D to indicate that the effect of NaCl was not due to changes in the osmolarity and that Na^+^ was the active cation.

### ATP-mediated calcium uptake in isolated acidocalcisomes and permeabilized cells

Calcium uptake into acidocalcisomes is mediated primarily by a Ca^2+^/H^+^ exchanging ATPase. Evidence for ATP-mediated calcium uptake into acidocalcisomes in both permeabilized cells and isolated organelles has been obtained in trypanosomatids [12], [18], [26]. Additionally, Ca^2+^-ATPases have been cloned and localized to acidocalcisomes by immunological methods in both trypanosomes and *T. gondii* [3], [27]. However, our previous attempts to demonstrate calcium uptake directly in *T. gondii* have not been successful. Using organelles collected using the refined methodology presented in this paper, we reexamined this issue.

**Figure 5:**
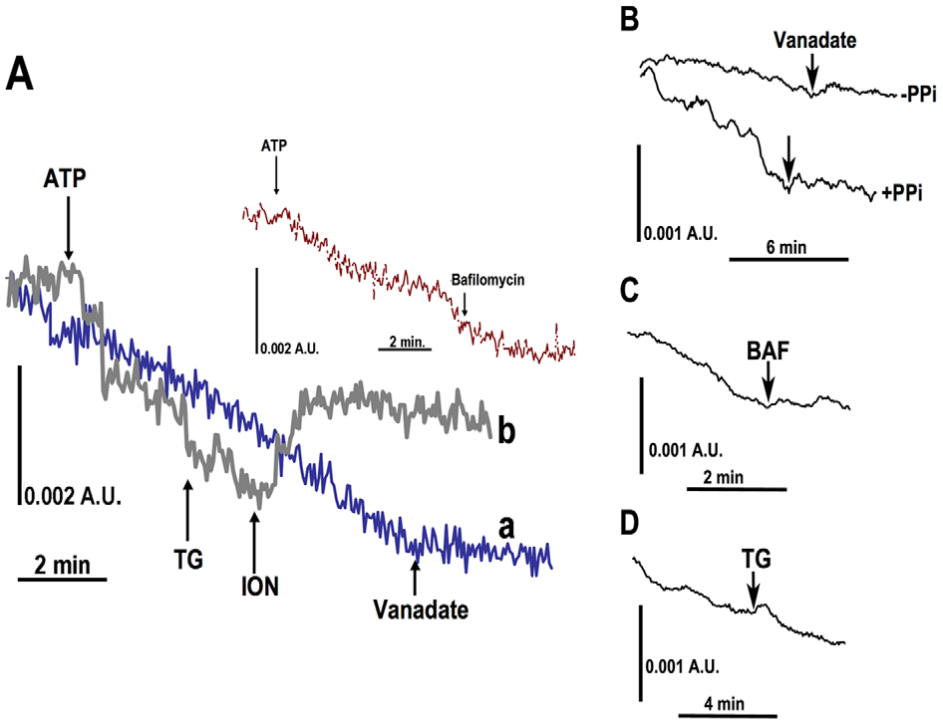
Calcium transport by acidocalcisomes. **A,** Detection of ATP-mediated calcium uptake activity in permeabilized tachyzoites (**A**) and isolated acidocalcisomes (**B–D**). Changes in calcium were followed using the calcium-sensitive dye Arsenazo III. Reaction mixture and conditions are described under “Materials and Methods”. ATP (1 mM), thapsigargin (TG, 1 µM), ionomycin (ION, 1 µM), bafilomycin A_1_ (1 µM), and vanadate (40 µM) were added where indicated. The noise is high in these tracings because of the absorbance scale needed to detect changes. AU, arbitrary absorbance units. Experiments shown in **A–D** were qualitatively similar in at least 3 different experiments.

In the presence of the mitochondrial inhibitors oligomycin and antimycin A, ATP-mediated calcium uptake in digitonin-permeabilized tachyzoites was readily observed as a decrease in the ratiometric absorbance of the calcium-sensitive dye Arsenazo III (Fig. 5A). This calcium uptake was not further inhibited by the addition of the sarcoplasmic-endoplasmic reticulum Ca^2+^-ATPase (SERCA) inhibitor thapsigargin and could be partially released by the calcium ionophore ionomycin (Fig. 5A, *trace b*). The uptake mechanism could be inhibited by the addition of bafilomycin A_1_, which inhibits the vacuolar-type H^+^-ATPase (Fig. 5A, *inset*). The proton dependence of the uptake mechanism is consistent with the previously described Ca^2+^/H^+^-exchanging activity of the acidocalcisomal Ca^2+^-ATPase. Finally, the uptake could be inhibited by the general ATPase inhibitor vanadate (Fig. 5A, *trace a*), confirming that calcium influx is mediated by a P-type Ca^2+^-ATPase activity. The concentrations of inhibitors used were shown before to be active against the P-type Ca^2+^-ATPase (vanadate), the V-H^+^-ATPase (bafilomycin A_1_), and the SERCA-type Ca^2+^-ATPase (thapsigargin) [2], [5], [17] of *T. gondii*. Similar qualitative results were obtained in both wild type RH (Fig. 5A) and TgVP1-overexpressing cell line (data not shown). Taken together, these results are consistent with the presence of a non-mitochondrial, non-ER Ca^2+^/H^+^-ATPase in *T. gondii*.

We performed similar experiments on isolated acidocalcisome fractions. In the continuous presence of the mitochondrial inhibitors oligomycin and antimycin, an ATP-mediated calcium uptake could readily be demonstrated (Fig. 5B–D). This uptake was more pronounced if acidocalcisomes were pre-incubated with PP_i_ before the addition of ATP and was inhibited by vanadate (Fig. 5B). This is consistent with our observations (Fig. 3G) and previous work [12] that many acidocalcisomes lose their acidity upon isolation. Reconstitution of the proton gradient by the vacuolar H^+^-pyrophosphatase upon the addition of PP_i_ enhanced calcium uptake (Fig. 5B), and addition of bafilomycin A_1_ was inhibitory (Fig. 5C). This provides further confirmation that calcium uptake occurs through a Ca^2+^/H^+^-ATPase-mediated mechanism. Finally, addition of thapsigargin did not inhibit calcium uptake (Fig. 5D). Taken together, these results confirm a Ca^2+^/H^+^-ATPase activity in isolated acidocalcisomes of *T. gondii* that is not due to contamination by ER or mitochondrial elements.

## Discussion

Based on analogy to the definitive work on acidocalcisomes in trypanosomatids [1], several studies have provided morphological, spectroscopic [20] and biochemical [2], [5] evidence for the presence of these acidic calcium and polyphosphate-rich organelles in *T. gondii* as well. However, several features of the studies in trypanosomatids could not be reproduced in *T. gondii*. Most importantly, it was not possible to demonstrate proton or calcium uptake in isolated organelles.

In this paper we describe a refined method for isolating acidocalcisomes from *T. gondii* tachyzoites that produces larger quantities of material for analysis. Importantly, this method results in a fraction that is enriched in markers for acidocalcisomes, such as the vacuolar H^+^-pyrophosphatase and long-chain poly P, without significant contamination with markers for other organelles. Organelles obtained using this method exhibit typical acidocalcisome morphology when examined by electron microscopy and stain intensely for poly P.

Using isolated acidocalcisomes obtained from this procedure, we have been able to demonstrate for the first time both ATP- and PP_i_ -mediated proton uptake activities which are sensitive to the inhibitors bafilomycin A_1_ and AMDP, respectively. These results are consistent with the presence of the vacuolar-type H^+^-ATPase and H^+^-pyrophosphatase in acidocalcisomes of *T. gondii*, which are central to acidocalcisome function in other organisms [1]. Additionally, following acidocalcisomal acidification, the proton gradient could be released by the addition of sodium or calcium, consistent with the presence of Ca^2+^/H^+^ and Na^+^/H^+^ exchange systems, as described for other organism [13], [18]. We also detected Na^+^/H^+^ and Ca^2+^/H^+^ exchange activities in our PLV fractions and this is not unexpected because there is evidence that both organelles interact [17]. The PLV is a highly dynamic organelle and it appears to fuse with acidocalcisomes. It is quite possible that membrane components are translocated from one to the other compartment, as is the case for the TgVP1 and possibly the V-H^+^-ATPase.

Similarly, we have been able to demonstrate ATP-mediated calcium uptake in both permeabilized cells and isolated organelles. This activity is not due to mitochondrial or ER-mediated uptake, as it occurred in the presence of inhibitors of these systems. Several lines of evidence, including the release of protons by calcium, the enhancement of uptake by pre-incubation with PP_i_ and the inhibition of uptake by bafilomycin A_1_, suggest that the activity detected is indeed a Ca^2+^/H^+^-ATPase, as described for other organisms [1].

In summary, we have described a refined method for isolating acidocalcisomes from *T. gondii* tachyzoites that results in sufficient quantity and purity for biochemical and physiological analysis. The relative ease of the method will allow both for more extensive work on ion and calcium homeostasis in *T. gondii* as well as for the development of research in new directions, including proteomics.

## Supporting Information

Figure S1 **Visualization of acidocalcisomes and spatial mapping of elemental distribution by whole-cell electron microscopy.** Acidocalcisomes are clearly identified as round, electron dense structures seen throughout the cell in **A. B-I,** specific elements are mapped from the cells identified in **A.** Scale bar  =  0.5 µm.
(PDF)
