# Supplementary material for: Calcium Uptake and Proton Transport by Acidocalcisomes of Toxoplasma gondii
Source: PLoS One. 2011 Apr 25;6(4):e18390. doi: 10.1371/journal.pone.0018390 (PMC3081817; doi:10.1371/journal.pone.0018390)
Supplement: Figure S1 — Visualization of acidocalcisomes and spatial mapping of elemental distribution by whole-cell electron microscopy. Acidocalcisomes are clearly identified as round, electron dense structures seen throughout the cell in A. B-I, specific elements are mapped from the cells identified in A. Scale bar = 0.5 µm. (PDF) [file pone.0018390.s001.pdf]

Calcium uptake and proton transport by acidocalcisomes of *Toxoplasma gondii.*

Rohloff et al.

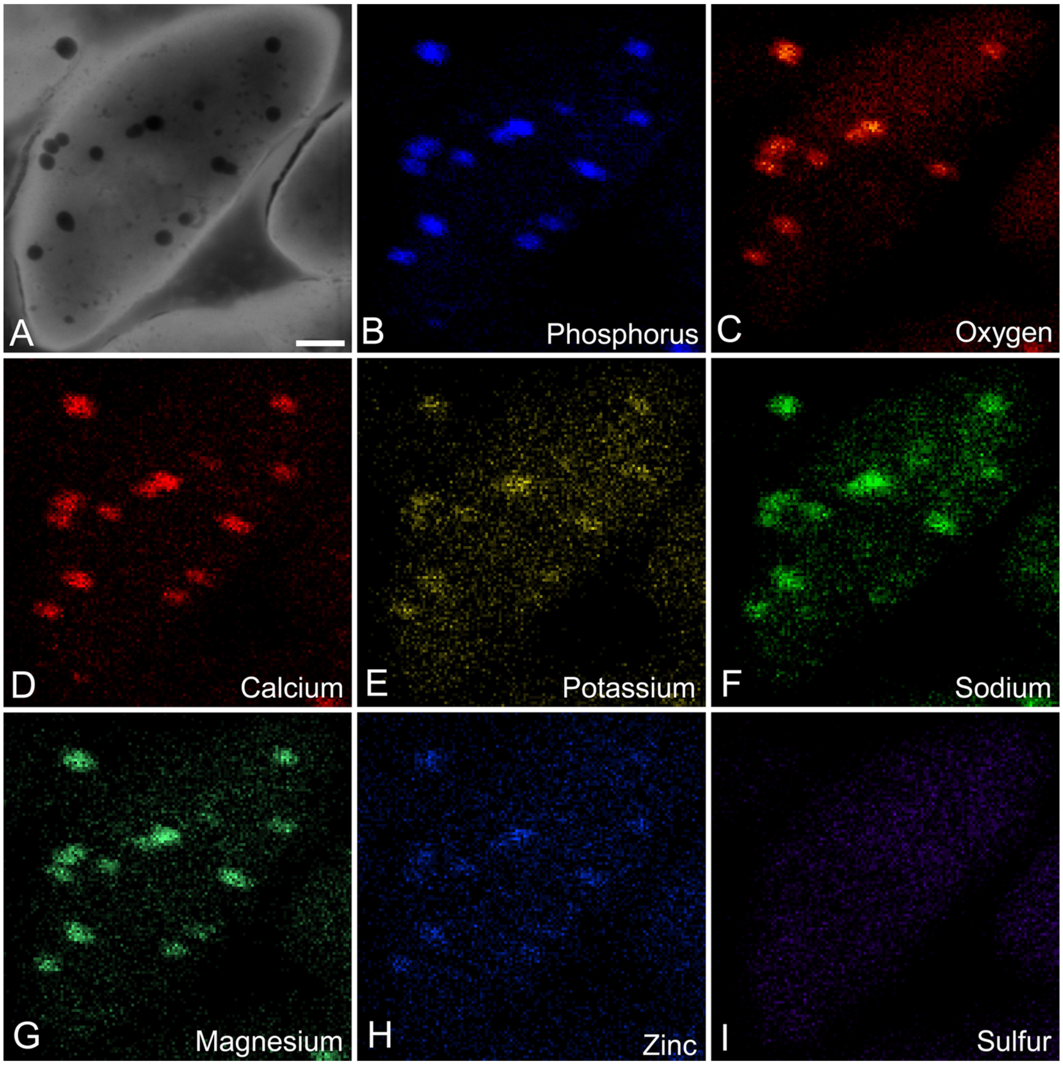

Figure S1. Visualization of acidocalcisomes and spatial mapping of elemental distribution by whole-cell electron microscopy. Acidocalcisomes are clearly identified as round, electron dense structures seen throughout the cell in A. B-I, specific elements are mapped from the cells identified in A. Scale bar = 0.5 µm.
